# Nanosecond Q-Switched 1064/532 nm Laser to Treat Hyperpigmentations: A Double Center Retrospective Study

**DOI:** 10.3390/clinpract11040086

**Published:** 2021-09-23

**Authors:** Steven Paul Nisticò, Giovanni Cannarozzo, Eugenio Provenzano, Federica Tamburi, Gilda Fazia, Mario Sannino, Francesca Negosanti, Ester Del Duca, Cataldo Patruno, Luigi Bennardo

**Affiliations:** 1 Department of Health Sciences, Magna Graecia University, 88100 Catanzaro, Italy; federica.tamburi@gmail.com (F.T.); gildafazia@gmail.com (G.F.); ester.delduca@gmail.com (E.D.D.); cataldopatruno@libero.it (C.P.); luigibennardo10@gmail.com (L.B.); 2 Unit of Dermatology, Tor Vergata University, 00133 Rome, Italy; drcannarozzo@gmail.com (G.C.); dr.mariosannino@gmail.com (M.S.); 3 Unit of Dermatology, Mariano Santo Hospital, 87100 Cosenza, Italy; eprovenzano0@gmail.com; 4 Dermatologic Center, 40100 Bologna, Italy; francesca.negosanti@gmail.com

**Keywords:** Q switched laser, benign hyperpigmentations, melasma, solar lentigo

## Abstract

(1) Benign hyperpigmentations are a common problem in cosmetic dermatology. Melasma, solar lentigo, and other acquired hyperpigmentations represent an aesthetic issue for an increasing number of patients. The gold standard in managing this condition is currently 1064/532 nanometers (nm) Q-Switched lasers. This study reports our experience on the use of a Q-switched laser with a nanosecond pulse to treat these conditions. (2) Methods: A total of 96 patients asking for benign hyperpigmentation removal were consecutively enrolled at the Magna Graecia University of Catanzaro and Tor Vergata University of Rome. Treating parameters were the following: 1064 nm with a pulse duration of 6 nanoseconds (ns) for dermic lesions and 532 nm with 6 ns for epidermal ones. Up to five treatments with a minimum interval between laser treatments of thirty days were performed. A follow-up visit three months after the last session assessed patient satisfaction with a Visual Analogue Scale (VAS). Two blinded dermatologists assessed the cosmetic result using a five-point scale comparing pictures before treatment and at follow-up. (3) Results: 96 patients were included; 47 participants were women (49.0%) and 49 men (51.0%). The mean reported age was 50.0 ± 17.3 years. All patients reached a good to complete hyperpigmentation removal at the dermatological evaluation with a mean VAS score of 8.91 ± 1.07. (4) Conclusions: Q Switched 1064/532 nm laser may be considered the gold standard treatment for benign hyperpigmentations. Our results confirm the literature findings on the effectiveness of these devices.

## 1. Introduction

Hyperpigmentation may be defined as the darkening of the skin due to a melanin deposit in the epidermis or dermis. Removal of such lesions for cosmetic reasons is a procedure that is becoming more and more common in dermatologic practices [1]. Various methods have been proposed to manage these melanoses, such as surgery, chemical ablation, and clarifying creams [2].

Lasers traditionally used to treat exophytic lesions, such as CO_2_ lasers, have been initially proposed, especially in fractional mode, exploiting their ability to convey energy in the superficial layers of the skin [3,4]. These devices are associated with a more considerable risk of scarring and pigmentation changes [5,6]. For this reason, lasers selectively acting on chromophores have been proposed and have obtained better results. Among these devices, different studies indicate that Q-switched lasers deliver high energies in the order of nano or picoseconds and act selectively on the melanin chromophore sparing surrounding tissues, and therefore may be considered as the most effective and safe treatment for benign hyperpigmentations [7,8,9].

In this study, we assessed the safety and effectiveness of a Q-switched 1064/532 nm laser with a nanosecond pulse range on common dermal and epidermal hyperpigmentations.

## 2. Materials and Methods

Patients with benign hyperpigmentations were enrolled in this open study conducted in two different Italian dermatological clinics, Magna Graecia University (Catanzaro, Italy) and Tor Vergata University (Rome, Italy), from 1 January 2019 to 30 December 2019. Reported exclusion criteria were the following: hypersensitivity to light (visible and near-infrared); medication known to increase sensitivity to light; therapies with anticoagulants and/or immunosuppressants; pregnancy or nursing; personal or family history of skin cancer; sun exposure in the three weeks before treatment (for any skin type); previous hyperpigmentation removal treatment; gold-containing medication; recent exfoliation treatments, surgical treatments and past skin disorders (including keloids). All patients signed informed consent on the risk of the procedure. Lesions were clinically classified as epidermal or dermal lesions.

Patients included in the study underwent treatment with a Q-switched 1064/532 nm laser system (Pico, Deka M.E.L.A., Calenzano, Italy), which provides ultrashort pulses to achieve selective photothermolysis of the target (melanin) with minimum thermal damage to surrounding biological structures. Treating parameters were the following: 1064 nm, up to 7 J/cm^2^ with a pulse duration of 6 nanoseconds (ns) for dermic lesions and 532 nm up to 2.5 J/cm^2^ and 6 ns for epidermal ones. Melasma was treated using the following parameters: spot 4 mm, fluence 1.5–2 J/cm^2^, 1–2 Hz. Laser was performed at single pulse, and multiple passes were made until the whitening of the treated lesion. Laser sessions were performed at least 30 days apart or until complete recovery of the skin from previous treatment. Final evaluation and follow-up visits took place three months after the last laser treatment; the clinical endpoint for treatments was the complete removal of benign hyperpigmented lesion(s).

**Figure 1:**
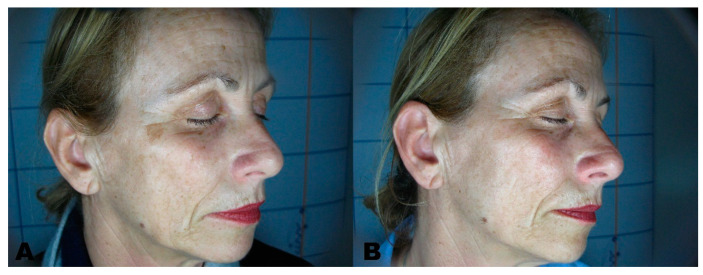
(**A**) Solar Lentigo before treatment. (**B**) region three months after the last session.

**Figure 2:**
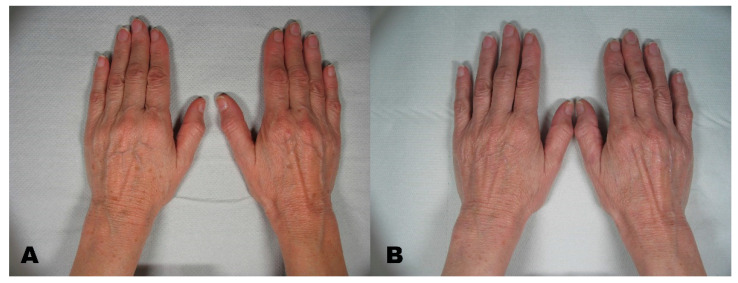
(**A**) Multiple lentigo before treatment. (**B**) region three months after the last session.

**Figure 3:**
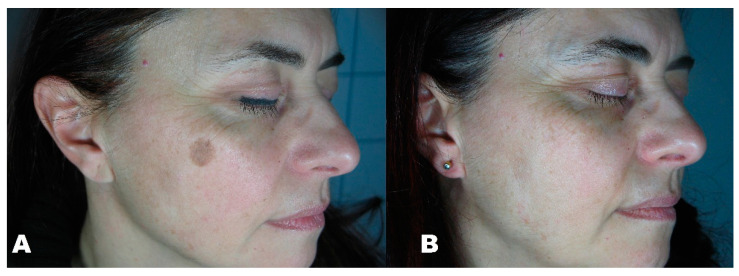
(**A**) Solar lentigo before treatment. (**B**) region three months after the last session.

**Figure 4:**
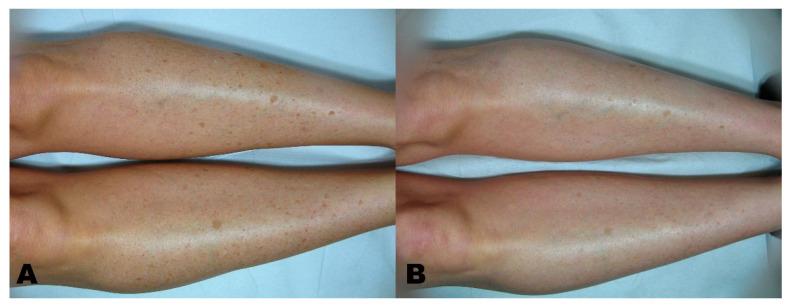
(**A**) Hypermelanosis before treatment. (**B**) region three months after the last session.

**Figure 5:**
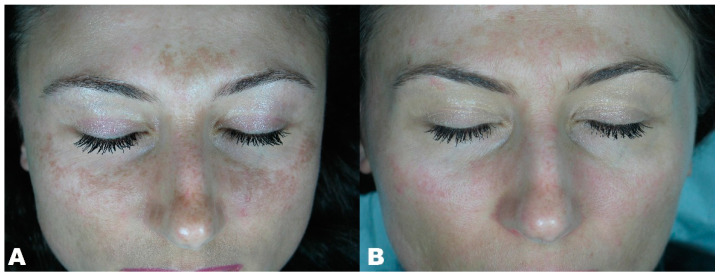
(**A**) Melasma before treatment. (**B**) Melasma three months after treatment.

Before the first session, clinical photographic documentation was carried out and repeated three months after the last session. Pictures were taken using the same camera (Nikon 5600d, Nikon Corporation, Minato City, Tokyo, Japan) and parameters, the same shooting settings, a twin flash, and the same ambient light (Figure 1, Figure 2, Figure 3, Figure 4 and Figure 5).

Two independent dermatologists evaluated the pictures, providing the final result in hyperpigmentation removal with a score in a 5-point scale (0–20% removal = 0; 20–40% removal = 1; 40–60% removal = 2; 60–80% removal = 3; 80–100% removal = 4).

A Visual Analogue Scale (VAS) from 1 to 10 was administered to the patients at the three-month follow-up to measure patient satisfaction.

Data analysis (mean, standard deviations, and rate calculations) was performed using Statistica 14.0 (TIBCO Software, Palo Alto, CA, USA).

## 3. Results

In this retrospective study 96 patients were included; 47 participants were women (49.0%) and 49 men (51.0%). The mean age was 50.0 ± 17.3 years. Different Fitzpatrick skin types could be observed since 38 patients showed type II skin (39.6%), 28 type III (29.2%), and 30 type IV (31.3%). In 92 cases (95.8%), benign hyperpigmentations were limited to the epidermis; only 4 (4.2%) were deeper, reaching the dermal stratum. The study population presented most lesions on the face (*n* = 72–75.0%), while 12 lesions (12.5%) were treated on the trunk and 12 on the hands, respectively.

Up to five laser sessions were necessary to achieve the clinical endpoint of complete hyperpigmentation removal; the mean number of laser sessions was 1.2 ± 0.7. In general, hyperpigmented lesions could be safely and effectively removed in 1–2 treatments, except deeper, dermal lesions requiring three to five laser sessions.

The dermatologist blinded evaluation reported the maximum score in 82 cases of epidermal lesions (89%) and two dermal lesions (50%). The mean score reported overall was 3.85 ± 0.41. Epidermal lesions reported a score of 3.88 ± 0.36, while dermal lesions reported 3.25 ± 0.96, showing an overall less efficacy in treating deeper lesions.

The mean visual analog scale administered to the patients three months after the last session was 8.91 ± 1.07; patients treated for epidermal lesions had a slightly higher score (9 ± 0.94), while patients with dermal lesions had a lower score (6.75 ± 1.71).

No serious adverse event occurred, treatment was well tolerated by all patients. Two patients with dermal hyperpigmentations experienced purpura local vascular recall as a side effect, spontaneously resolving in some days. After laser treatment on epidermal pigmented lesions, the majority of patients experienced transient perilesional erythema and edema, solved in 1–2 days, sometimes accompanied by itching; regular progress after treatment included treated lesions getting darker and covered by a flake/crusty formation, which exfoliated and turned into transient hypopigmentation until complete healing (no more visible effect) within 30 days.

Patient characteristics and subgroups division are reported in Table 1 and Table 2.

## 4. Discussion

Hypermelanosis are characterized by the accumulation of melanin at different levels of the skin. The mechanism of the Q-Switched laser primarily relies on the photomechanical effect to destroy the targeted tissue, accompanied by a diminished photothermal effect [10,11,12]. Hypermelanoses should be treated with lasers only when malignancy risk is excluded. For this reason, management of melanocytic nevi with light sources is usually contraindicated. However, various hyperpigmentation disorders of the skin may be treated with lasers [13,14,15]. These lesions may be classified into epidermal pigmented lesions (such as actinic lentigos, lentigo simplex, ephelides, café au lait spots, linear hyper melanosis, Spilus, and Becker spots) and dermal pigmented lesions (such as melasma, post-inflammatory hyperpigmentation, acquired and congenital dermal melanocytosis). Actinic lentigos are sun-induced hyperpigmentations that do not darken or increase in number after sun exposure [16].

Lentigines are light to dark brown macules frequently arising during childhood in sun-exposed areas. Freckles are small pigmented macules with irregular borders, visible after solar exposure and fading in winter or absence of sun radiations. Due to the superficial distribution of melanin, all these epidermic lesions are treated with the Q-switched laser at 532 nm wavelength [17].

Café au lait spots are macules with dimensions ranging from a couple of millimeters to various centimeters present since birth or appearing in the first years of the patient’s life. They do not have any relationship with sun exposure. These lesions are usually treated with Q-switched 1064 nm lasers with good results. Melasma is a tan or dark, bilateral, blotchy, brownish facial pigmentation. It is probably the most complex hyperpigmentary disorder with a poorly understood etiology. Although Q-Switched 1064 nm laser seems to have better results than other types of lasers, results remain controversial [18,19,20].

Acquired hyperpigmentations and congenital ones are usually treated with 1064 nm Q-switched lasers; acquired pigmentation tends to have better results than the congenital counterpart [21,22,23]. Q-switched laser treatment is usually well tolerated. Side effects may include pain, erythema, edema, pinpoint bleeding, crusting, blistering, scarring, and postinflammatory hyperpigmentation. Our study’s results suggest increased effectiveness of lasers in the epidermal lesions, with better results when treating the extremities or the trunk, and lighter phototypes [24,25,26]. Digital skin analysis devices, especially when performing melanin biometric measurements, may be very useful in better scoring the improvement of the patient. These devices use reflectance mapping of several different light wavelengths to precisely quantify the presence of melanin in the various layers of the skin, making the measurement of cosmetic results as standardized as possible. Given the high cost of these devices, they are unfortunately not always available.

The limitations of our study include the relatively small sample size, no use of digital skin analysis devices, and histological comparison before and after treatment (although performing a histological examination in patients seeking cosmetic results may be tricky).

## 5. Conclusions

Our study confirms results previously obtained in medical literature, highlighting Q-Switched laser treatments as the gold standard for hyperpigmentations. In our cohort of patients, lighter phototypes, lesions located on the trunk or limbs, and superficial melanosis better responded to the treatment. On the contrary, subjects with darker phototypes, facial lesions, and deeper pigmentation disorders responded poorly to the treatment, and a large number of sessions were required to achieve a satisfactory result. No severe side effects were reported; only minor events such as purpura and superficial crusting occurred occasionally. 

In conclusion, further prospective and comparative studies with a more significant number of patients would help to confirm our results.

## Figures and Tables

**Table 1:** Demographic data of included patients.

Patient No.	96
Female (%)	47 (49.0%)
Male (%)	49 (51.0%)
Mean Age ± SD [years]	50.0 ± 17.3
Age range [years]	19–77
*Fitzpatrick phototype:*	
II (%)	38 (39.6%)
III (%)	28 (29.2%)
IV (%)	30 (31.3%)
*Hyperpigmentation depth:*	
Epidermal (%)	92 (95.8%)
Dermal (%)	4 (4.2%)
*Hyperpigmentation location:*	
Face (%)	72 (75.0%)
Hand (%)	12 (12.5%)
Trunk (%)	12 (12.5%)

**Table 2:** Clinical parameters and removal scores in different subgroups.

	Mean No. Sessions ± SD	No. Sessions Range	Dermatologist Evaluation	Patient VAS Score
All patients	1.2 ± 0.7	1–5	3.85 ± 0.41	8.91 ± 1.07
*Fitzpatrick phototype:*				
II	1.3 ± 0.9	1–5	3.84 ± 0.44	9.10 ± 0.96
III	1.1 ± 0.3	1–2	4	9.07 ± 0.26
IV	1.3 ± 0.8	1–5	3.73 ± 0.52	8.34 ± 1.45
*Hyperpigmentation depth*				
Epidermal	1.1 ± 0.3	1–2	3.88 ± 0.36	9 ± 0.94
Dermal	4.3 ± 1.0	3–5	3.25 ± 0.96	6.75 ± 1.71
*Hyperpigmentation location*				
Face	1.3 ± 0.8	1–5	3.82 ± 0.45	8.78 ± 1.17
Hand	1.0 ± 0.0	1	4	9.42 ± 0.51
Trunk	1.0 ± 0.0	1	3.91 ± 0.29	9.17 ± 0.58

## Data Availability

Data are available from the corresponding author upon reasonable request.
